# Endogenous and viral microRNAs in nasal secretions of water buffaloes (*Bubalus* *bubalis*) after *Bubaline* *alphaherpesvirus* *1* (BuHV-1) challenge infection

**DOI:** 10.1186/s13567-023-01175-9

**Published:** 2023-06-05

**Authors:** Cristina Lecchi, Fabrizio Ceciliani, Stefano Petrini, Giovanna Cappelli, Carlo Grassi, Anna Balestrieri, Giorgio Galiero, Ester DeCarlo, Gaspare Salvi, Filippo Panzeri, Chiara Gini, Alessandra Cafiso, Alessandro Agazzi, Alessandra Martucciello

**Affiliations:** 1 Università Degli Studi Di Milano, 26900 Lodi, Italy; 2 National Reference Centre for Hygiene and Technologies of Water Buffalo Farming and Productions (CReNBuf), Istituto Zooprofilattico Sperimentale del Mezzogiorno, Via Salute 2, 80055 Portici, NA Italy; 3 National Reference Centre for Bovine Infectious Rhinotracheitis (IBR), Istituto Zooprofilattico Sperimentale Dell’Umbria E Delle Marche “Togo Rosati”, 06126 Perugia, PG Italy

**Keywords:** *Bubaline alphaherpesvirus 1* (BuHV-1), microRNA, Water buffaloes, Molecular biomarkers, Challenge infection

## Abstract

*Bubaline alphaherpesvirus 1* (BuHV-1) is a pathogen of water buffaloes responsible for economic loss worldwide. MicroRNAs (miRNAs) regulate gene expression produced by alphaherpesviruses and hosts. This study aimed at (a) unravelling the ability of BuHV-1 to produce miRNAs, including hv1-miR-B6, hv1-miR-B8, hv1-miR-B9; (b) measuring the host immune-related miRNAs associated to herpesvirus infection, including miR-210-3p, miR-490-3p, miR-17-5p, miR-148a-3p, miR-338-3p, miR-370-3p, by RT-qPCR; (c) identifying candidate markers of infection by receiver-operating characteristic (ROC) curves; (d) exploiting the biological functions by pathway enrichment analyses. Five water buffaloes BuHV-1 and *Bovine alphaherpesvirus 1* (BoHV-1) free were immunized against Infectious Bovine Rhinotracheitis (IBR). Five additional water buffaloes served as negative controls. All animals were challenged with a virulent wild-type (wt) BuHV-1 via the intranasal route 120 days after the first vaccination. Nasal swabs were obtained at days (d) 0, 2, 4, 7, 10, 15, 30, and 63 post-challenge (pc). The animals of both groups shed wt BuHV-1 up to d7 pc. Results demonstrated that (a) miRNAs produced by the host and BuHV-1 could be efficiently quantified in the nasal secretion up to d63 and d15 pc, respectively; b) the levels of host and BuHV-1 miRNAs are different between vaccinated and control buffaloes; c) miR-370-3p discriminated vaccinated and control animals; d) host immune-related miRNAs may modulate genes involved in the cell adhesion pathway of the neuronal and immune system. Overall, the present study provides evidence that miRNAs can be detected in nasal secretions of water buffaloes and that their expression is modulated by BuHV-1.

**Supplementary Information:**

The online version contains supplementary material available at 10.1186/s13567-023-01175-9.

## Introduction

*Bubaline alphaherpesvirus 1* (BuHV-1), a member of the *Alphaherpesviridae* family, is an important pathogen of water buffaloes, causing significant economic losses to the dairy industry worldwide. Acute infection begins in the mucosal epithelium and is followed by high levels of virus shedding. Infection with BuHV-1 can lead to several clinical signs, including conjunctivitis, pneumonia, genital disorders, abortions, and respiratory disease, promoting immune suppression [1, 2]. BuHV-1 is closely related to *Bovine alphaherpesvirus 1* (BoHV-1). The two viruses share their antigenic properties [3].

MicroRNAs (miRNAs) are short, single-stranded non-coding RNAs able to influence gene expression by inhibiting messenger RNA (mRNA) translation or inducing mRNA degradation [4]. MiRNAs are produced by mammalian cells regulating several pathophysiological processes [5], and host immune response. By trying to overcome the cellular intrinsic and extrinsic anti-viral immunity, the viruses, particularly the mammalian alphaherpesviruses examined to date, can micromanage virus- and host-encoded miRNAs cellular activities [6, 7]. The first evidence that mammalian alphaherpesviruses encode miRNAs was reported in 2005 [8]. Further data on miRNAs of six mammalian alphaherpesviruses—herpes simplex virus (HSV)-1 and -2, varicella-zoster virus (VZV), herpes B virus, BoHV-1, and pseudorabies virus (PRV, suid herpesvirus 1)—have been reported [6, 9–13]. The genome of BoHV-1 encodes at least 10 miRNAs [9], three of which, namely bhv1-miR-B6, bhv1-miR-B8, and bhv1-miR-B9, can modulate in vitro BoHV-1replication. Using in silico analysis, Kanokudom et al. [14] identified as potential binding sites for bhv1-miR-B6, Bhv1-miR-B8, and Bhv1-miR-B9 the BICP22 transcript, potentially interfering with the normal BoHV-1 replication cycle. Glazov et al. identified a potential mRNA target, the gene-infected cell protein 0 (ICP0), potentially hampering the progression of infection [9]. The scope of the present study was to characterize the behaviour of some of the alphaherpesvirus-related microRNAs, including those produced by BuHV-1 and those produced by the host. In detail, this study aimed to (a) unravel the ability of BuHV-1 to produce miRNAs, namely bhv1-miR-B6, bhv1-miR-B8, bhv1-miR-B9, previously identified as pivotal players in BoHV-1 replication; (b) quantify the BuHV- and host immune-related miRNAs (miR-210-3p, miR-490-3p, miR-17-5p, miR-148a-3p, miR-338-3p, miR-370-3p—previously associated to the host response against herpesvirus) in the nasal secretion of water buffaloes experimentally infected with BuHV-1 by RT-qPCR; (c) point out candidate markers of infection by receiver-operating characteristic (ROC) curves; (d) exploit the biological functions by pathway enrichment analyses to get insights into the pathogenesis of herpesvirus infection.

## Materials and methods

### BuHV-1

The wild-type (wt) 799,787 MT strain of BuHV-1 isolated during a BuHV-1 outbreak in a buffalo herd in south Italy was used in this study [GenBank KF679678.1]. The strain was used at the second passage on Madin-Darby Bovine Kidney (MDBK) cell cultures at a titre of 1.5 × 10^8.00^ median tissue culture infectious dose (TCID_50_/mL).

### Immunisation protocol

This study used two commercial gE-deleted marker vaccines (A, B). Vaccine A was administered at 3 months of age (time 0), and then a second dose was inoculated after one month (30 post-vaccination days, pvd). Vaccine B was injected at 10 months of age (210 pvd), and then a second dose was injected at 11 months of age (240 pvd). Vaccine A was injected intranasally (i.n.); otherwise, vaccine B was administered intramuscularly (i.m.) into the neck muscle. Each dose of the vaccine was injected in a volume of 2 mL [15].

### Experimental design

Ten water buffaloes without neutralizing antibodies (NA) to BuHV-1 and BoHV-1 were enrolled. These animals were selected from a water buffalo herd in Southern Italy (Calabria region). In the herd, no vaccination against BoHV-1 and no history of respiratory disease had been detected in the last 5 years. After selection, animals were transferred to an experimental facility for large animals at the Istituto Zooprofilattico Sperimentale del Mezzogiorno in Southern Italy (Campania region). The animals were fed twice daily with hay, concentrated fattening feed, and water ad libitum.

All experimental procedures followed European legislation protecting of animals used for scientific purposes [Directive 2010/63]. Furthermore, the Italian Ministry of Health approved the experiments under authorization number 859/2017-PR.

The number of animals in each group was determined through the sampling procedure envisaged in an experimental clinical study to compare proportions in terms of superiority, with an error of 1% and a study power of 80%.

Animals were divided into two groups of five animals each. The water buffaloes in the first group (A) were immunized with the protocol described in the “Immunisation Protocol” section. The second group (B) served as an unvaccinated control group. Animals from each group were housed in separate pens.

Two hundred seventy days after the first vaccination (d270 pvd; 12 months of age), all water buffaloes were subjected to challenge infection with a wt BuHV-1 strain. Each animal received 5 × 10^6.74^ TCID_50_ /mL administered via the intranasal route.

Water buffaloes were observed for 63 days pc, and rectal temperatures were taken daily. Fever was confirmed when the rectal temperature was greater than 38.2 °C [15]. A veterinary practitioner constantly monitored any appearance of adverse reactions after vaccination protocol, both local (transient tissue reaction at the injection site; nasal hypersecretion) and systemic (hyperthermia, hypersensitivity). If these adverse reactions occur, the veterinary practitioner administered the necessary drugs to relieve animal suffering (anti-inflammatory, antipyretics, and cortisone).

On the day (d) of the first vaccination (time 0), at d30, d210, and d240 post-vaccination days (pvd), serum samples were collected from each water buffalo and tested for anti- BoHV-1 antibodies by ELISA tests. In particular, the IDEXX IBR gE Ab test and IDEXX IBR gB X3 Ab test were used to detect glycoprotein B (gB) or glycoprotein E (gE) of BoHV-1. These tests, developed for BoHV-1, cross-react with the glycoproteins (gB -gE) of BuHV-1 [16]. 

Furthermore, serum samples were collected from all water buffaloes at d0, d2, d4, d7, d10, d15, d30, and d63 post-challenge (pc). The samples were tested using the IDEXX IBR gE Ab test and IDEXX IBR gB X3 Ab test. In addition, throughout the experimental period, sera were assayed with In3Diagnostic Eradikit TM BoHV1-BuHV-1 discrimination kit. This test differentiates the gE of BoHV-1 from the gE of BuHV-1.

### Nasal swab collection

Nasal swabs were collected from each buffalo at d0, d2, d4, d7, d10, d15, d30, and d63 pc, placed in transport fluid Minimum Essential Medium (Euroclone, Milan, Italy), and used for BuHV-1 isolation and titration assays. Serial dilutions ranging from 10^–1^–10^–9^ of supernatants from each nasal swabbing were inoculated in a volume of 0.1 mL into three wells of a 24-well plastic plate containing monolayers of MDBK cell cultures grown in MEM. The cells were provided by Biobanking of Veterinary Resources (BVR), Brescia, Italy, and identified with the code BS CL63. After 60-min incubation at 37 °C in a 5% CO_2_ atmosphere, 1 mL of MEM enriched with 2% FCS (BioWhittaker Inc., Walkersville, MD, USA) was added to each well. The positive control was prepared from MDBK cell cultures infected with the wt 799,787 MT strain of BuHV-1. MDBK cell cultures free of BuHV-1 were used as a negative control.

The plates were incubated for 7 days at 37 °C in a 5% CO_2_ atmosphere and observed daily for the appearance of cytopathic effect (CPE). BuHV-1 titre was determined as previously described [17] and expressed as TCID_50_/mL. The BuHV-1 recovered from each positive sample was identified by PCR [1].

### Blood sample collection

Blood samples (approximately 9 mL from each animal) were collected from the coccygeal veins, using sterile vacuum tubes (Serum BD vacutainer®; 10 mL) without anticoagulant. Samples were transported to the laboratory under refrigerated conditions within 1 h of collection before testing and stored at –20 °C until further processing. Afterward, blood samples were thawed and centrifuged at 850 × *g* for 30 min at 4 °C to extract the serum for serological investigations.

### ELISA tests

Serum samples were tested using three commercial ELISA tests (IDEXX IBR gE Ab test, Maine, USA; IDEXX IBR gB X3 Ab, Maine, USA; In 3Diagnostic, Eradikit™ BoHV1/BuHV1 discrimination Kit) used in parallel to examine the collected sera following manufacturer’s protocols. Microplates were read using an automated plate reader, and the data were analysed using the Magellan software (Tecan AG, Männedorf, Switzerland).

### Small RNA extraction and RT-qPCR

Small RNAs were extracted from nasal secretion swabs using the microRNA Concentrator kit (A&A Biotechnology, Cat. No 035-25) following the manufacturer’s instructions. *Caenorhabditis elegans* miRNA cel-miR-39 (25 fmol final concentration) (Qiagen, Cat. No 219610) was synthetic spike-in control.

RNA concentrations were quantified using the NanoDrop ND-1000 spectrophotometer (NanoDrop Technologies). The reverse transcription was performed on 15 ng of small RNAs for all samples using the TaqMan Advanced miRNA cDNA Synthesis Kit (Applied Biosystems, Cat. No. A28007) following the manufacturer’s instruction.

The quantitative PCR (qPCR) reaction was performed following the minimum information for publication of quantitative real-time PCR experiments (MIQE) guidelines [18].

The selection of miRNAs was based on their ability to modulate the host immune response or be expressed by alphaherpesvirus [14]. The selected TaqMan Advanced miRNA assays (Life Technologies) included cel-miR-39-3p (assay ID 478293_mir), miR-210-3p (assay ID mmu481343_mir), miR-490-3p (assay ID rno481188_mir), miR-17-5p (assay ID 478447_mir), miR-148a-3p (assay ID 477814_mir), miR-92a-3p (assay ID 477827_mir), miR-423-5p (assay ID mmu481834_mir), miR-338-3p (assay ID rno480884_mir), miR-370-3p (assay ID 478326_mir). Since TaqMan Advanced miRNA assays were unavailable for bhv1-miR-B6-5p, bhv1-miR-B8-5p, and bhv1-miR-B9, they were custom-designed by ThermoFisher Scientific service starting from traditional assays ID 242856_mat, 242520_mat, and 242648_mat targeting BoHV-1. To evaluate the ability of custom-designed probes to recognize BuHV-1 miRNAs, namely hv1-miR-B6-5p, hv1-miR-B8-5p, and hv1-miR-B9, the probes were tested on miRNAs extracted from heat-inactivated (60 °C for 30 min) wt strain of BuHV-1. The BuHV-1 was used at the second passage (10^8.00^ TCID_50_/mL) on MDBK cell cultures. Briefly, after miRNAs extraction and reverse transcription from BuHV-1 wt strain, a qPCR was performed as described later. The obtained PCR products were then cloned into pGEM(R)-T Easy Vector System II (Cat. No. A1380, Promega, Madison, WI, USA) and Sanger sequenced, as previously described [19].

RT-qPCR was performed on CFX Connect Real-Time PCR Detection System (Biorad). The reaction included 7.5 µL of 2X TaqMan Fast Advanced Master Mix (Cat. No. 4444557), 0.75 µL of miRNA-specific TaqMan Advanced assay (20X), 1 µL of cDNA, and water to a final volume of 15 µL. The thermal profile was 50 °C for 2 min, 95 °C for 3 min, and 40 cycles of 95 °C for 15 s and 60 °C for 40 s. A geNorm analysis [20] was performed using Biogazelle’s qbase + software [21], identifying miR-423-5p and miR-92a-3p as suitable reference miRNAs with the lowest M value (< 1.5) and their arithmetic mean was used as the normalization factor. The relative expression was calculated using Bio-Rad CFX Maestro™ Software. miRNA expression is presented in terms of fold change using the 2^−ΔΔCq^ formula.

### miRNA target prioritization

The target genes of differentially expressed (DE)-miRNAs were predicted using MiRWalk 3.0 [22], as previously described [23]. The list of target genes predicted by the three tools (miRDB [24], miRTarBase [25], and Targetscan [26]) was included in further analysis, and functional mRNA enrichment was performed using DAVID (Database for Annotation, Visualization and Integrated Discovery) bioinformatics resource [27, 28] and biological pathways in the KEGG (Kyoto Encyclopedia of Genes and Genomes) [29] were examined for enrichment.

### Statistical analysis

All data are expressed as the mean ± SD. Differences between experimental groups were assessed by one-way analysis of variance (ANOVA) using the Statistical Analysis System software (SAS version 9.4; SAS Institute Inc., Cary, NC, USA). A MIXED procedure for repeated measurements was used with animals at the experimental unit, accounting for the effects of vaccination (vaccinated or not), time, and their interactions. *P* < 0.05 was considered statistically significant. Receiver-operating characteristic (ROC) analysis is a valuable tool for evaluating the performance of diagnostic tests that classify subjects into one of two categories (vaccinated and control animals). ROC analysis was carried out by plotting the true positive (sensitivity) versus the false positive (1-specificity), cut-off points were set to maximize the sum of sensitivity and specificity, and the associated area under the curve (AUC), where an area of 1 represents a perfect test and an area of 0.5 represents a worthless test, was used to confirm the diagnostic potency of each miRNA [30].

## Results

### Clinical response

During the application of the immunisation protocol, clinical signs or adverse reactions were observed in none of the immunized water buffaloes. Rectal temperatures remained within normal healthy range values and were similar to control values. After challenge infection, no clinical signs were observed in all immunised water buffaloes. Differently, in unvaccinated controls, on d2 pc, four animals showed nasal mucus discharge and lesions at the nasal mucosa consisting of pseudomembranes associated with mucopurulent exudate. These lesions were treated after d15 pc. In addition, the rectal temperatures increased to 39.0 °C from d2 to d7 pc in control animals.

### BuHV-1 shedding

After challenge infection, all animals shed wild-type BuHV-1 from d2 to d7 pc (Table 1). The mean titre of the BuHV-1 shed by animals from the vaccinated group on d2 pc was 10^1.80^ TCID_50_/mL. This titre was maintained at d4 pc and decreased by 0.2 logs units at d7 pc. The number of animals that shed BuHV-1 was constant in 2 water buffaloes. Differently, the mean titre of BuHV-1 shed by water buffaloes from the unvaccinated group on d2 pc was 10^3.25^ TCID_50_/mL. This titre decreased to 1.10 log units d7 pc. The number of water buffaloes that shed BuHV-1 was 4, 5, and 3, detected at d2, d4, and d7 pc, respectively (Table 1).

**Table 1:** **BuHV-1 isolation from water buffaloes immunized against BoHV-1 using an immunisation protocol with gE-deletion marker vaccines and challenge infected with wild-type BuHV-1**

Group	Virus isolation and titration after challenge infection on day ^a^							
	0	2	4	7	10	15	30	63
A	–	1.8 (2)^b^	1.8 (2)	1.6 (2)	N.I	N.I	N.I	N.I
B	–	3.25 (4)	3.0 (5)	2.2 (3)	N.I	N.I	N.I	N.I

A: Vaccinated group, B: Control group.

^a^Reciprocal value of the negative log of TCID_50_/ mL (group mean value).

^b^The number of water buffaloes from which the virus was isolated are shown in brackets; N.I., Not isolated.

### Serological responses

At d30, d60, d210, and d240 pvd, vaccinated animals were seropositive for gB- ELISA and negative for gE-ELISA BoHV-1-BuHV-1 discrimination Kit. Likewise, no seroconversion was detected in unvaccinated controls (Table 2).

**Table 2:** **Antibody response of water buffaloes immunized against BoHV-1 using an immunisation protocol with gE-deletion marker vaccines**

Group		Post-vaccination day (pvd)			
		0	30	210	240
A	gE-ELISA^a^	−	−	−	−
	gB-ELISA^b^	−	+	+	+
	ELISA^c^	−	−	−	−
B	gE-ELISA^a^	−	−	−	−
	gB-ELISA^b^	−	−	−	−
	ELISA^c^	−	−	−	−

*A* Vaccinated group, *B* Control group.

^a^IDEXX IBR gEAb test, MAINE, USA.

^b^IDEXX IBR gB X3 Ab, Maine, USA.

^c^IN3Diagnostic Eradikit™ BoHV1-BuHV1 discrimination kit, Turin, Italy.

After challenge infection in vaccinated animals, the gB-ELISA positivity persisted until the end of the experiments (Table 3). In addition, the positive signal for gE was detected only on d30 pc. At the same time, a seroconversion was detected for BuHV1 using the BoHV-1-BuHV-1 discrimination kit. In the control group, antibodies for gB were detected on d10 pc, and the ensuing gB seropositivity succeeded until the end of the experiment. In the same group, the seropositivity for gE ELISA was detected d30 pc. A positivity to BuHV-1 was detected using the BoHV-1-BuHV-1 discrimination kit d15 pc (Table 3).

**Table 3:** **Antibody response of water buffaloes immunized against BoHV-1 using an immunisation protocol with gE-deletion marker vaccines and challenge infected with wild-type BuHV-1**

Group		Post-challenge day (pcd)							
		0^*^	2	4	7	10	15	30	63
A	gE-ELISA^a^	−	−	−	−	−	−	+	+
	gB-ELISA^b^	+	+	+	+	+	+	+	+
	ELISA^c^	−	−	−	−	−	−	+ ^	+ ^
B	gE-ELISA^a^	−	−	−	−	−	−	+	+
	gB-ELISA^b^	−	−	−	−	+	+	+	+
	ELISA^c^	−	−	−	−	−	+ ^	+ ^	+ ^

^*^The day of challenge corresponding to 270 pvd; A, Vaccinated group; B, Control group.

^a^IDEXX IBR gE Ab test, MAINE, USA. This test detects antibodies to glycoprotein E (gE) of BoHV-1/BuHV-1.

^b^IDEXX IBR gB X3 Ab, Maine, USA. This test detects antibodies to glycoprotein B (gB) of BoHV-1/BuHV-1.

^c^IN3Diagnostic Eradikit™ BoHV-1-BuHV-1 discrimination kit, Turin, Italy. This test discriminates antibodies to glycoprotein E (gE) against BoHV-1 and BuHV-1. NA, + ^, positive to BuHV-1.

### Expression of miRNAs produced by the host and by BuHV-1

**Figure 1:**
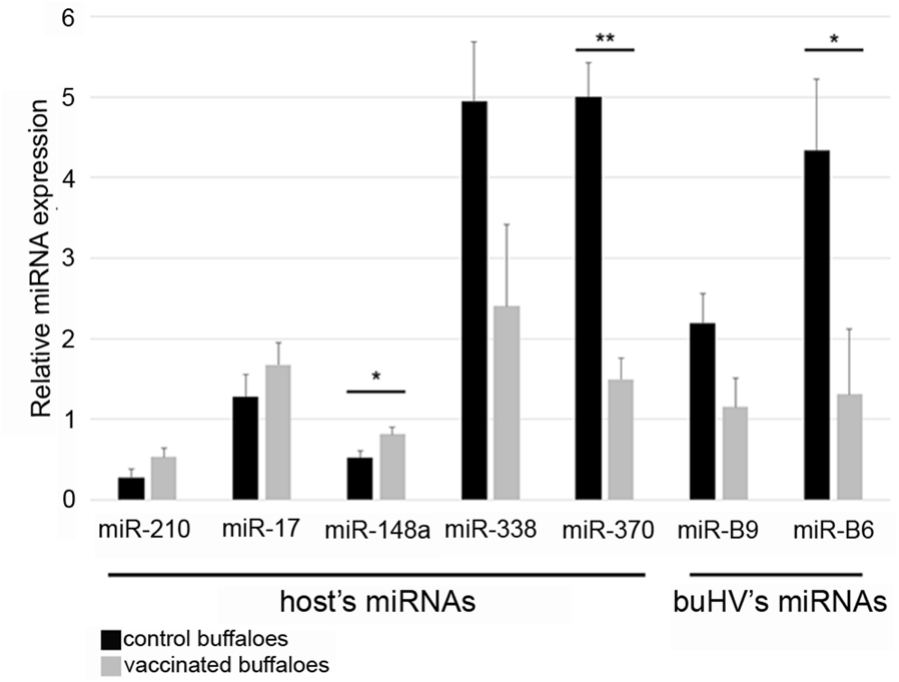
**Histograms of DE-miRNAs in control compared with vaccinated water buffaloes.** Significance was declared at *P* < 0.05 (*), *P* < 0.01 (**), and *P* < 0.001 (***).

Sanger sequencing demonstrated that the sequences of miRNAs bhv1-miR-B6-5p and bhv1-miR-B9 were conserved between BoHV-1 and BuHV-1 (Additional file 1). In contrast, the probe for hv1-miR-B8-5p did not recognize BuHV-1. RT-qPCR was performed on miRNAs extracted from the nasal swabs collected at 8-experimental time points. Quantification of bhv1-miR-B6-5p and bhv1-miR-B9 on water buffalo's nasal secretion demonstrated that the expression of bhv1-miR-B6-5p decreased in vaccinated compared to negative control animals (ratio 3.3; *P* = 0.035), while no modification occurred for bhv1-miR-B9 (Figure 1). Both bhv1-miR-B6-5p and bhv1-miR-B9 could be quantified until d15 pc. Then from d30 pc, their level drops off (data not shown).

Five (miR-210-3p, miR-17-5p, miR-148a-3p, miR-338-3p, miR-370-3p) out of six selected miRNAs were detected in nasal secretions, among which miR-148a-3p and miR-370-3p were differentially expressed (DE) between vaccinated and control water buffaloes (Figure 1). In detail, the level of miR-148a-3p increased (ratio = 1.55; *P* = 0.038), and the level of miR-370-3p decreased (ratio = 3.34; *P* = 0.005) in vaccinated compared to control animals. The effects of time (*P* = 0.0003) and time x vaccination (*P* = 0.0013) were significant only for miR-370-3p. Mir-490-3p was not detected in nasal secretions of water buffaloes.

### Diagnostic potential of miRNAs in nasal secretions

**Figure 2:**
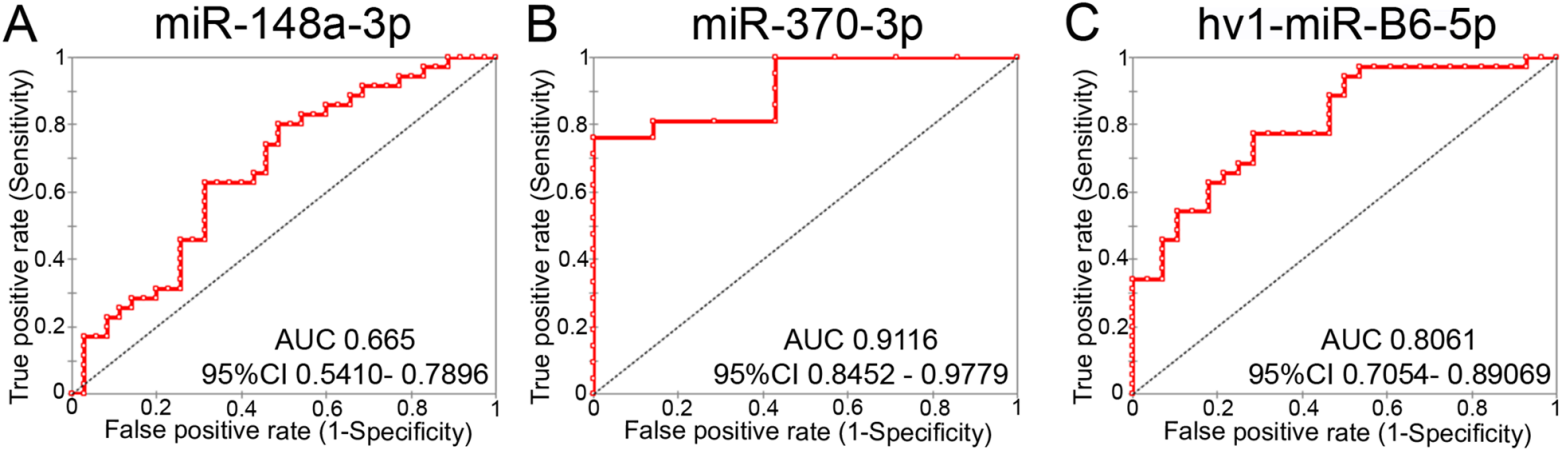
**Receiver-operator characteristics (ROC) curve comparing control and vaccinated water buffaloes.** **A** ROC of miR-148a-3p; **B** ROC of miR-370-3p; and **C** ROC of hv-miR-B6-5p. AUC, area under the curve; CI, confidence interval.

A ROC curve analysis was carried out to evaluate the diagnostic value of DE-miRNAs in nasal secretion (Figure 2). The associated area under the curve (AUC) was used to confirm the diagnostic potency of each miRNA. Cut-off points were set to maximize the sum of sensitivity and specificity (Table 4). The ability of DE-miRNAs to distinguish vaccinated from control animals is defined as diagnostic accuracy. It is measured by the AUC, where an area of 1 represents a perfect test, and an area of 0.5 represents a worthless test. The ability to discriminate vaccinated from control water buffaloes was excellent for miR-370-3p (AUC = 0.9116; 95% CI 0.8452–0.9779; *P* < 0.0001), bad for miR-148a-3p (AUC = 0.665; 95% CI 0.5410–0.7896; *P* < 0.009), and good for hv1-miR-B6-5p (AUC = 0.8061; 95% CI 0.7054–0.9069; *P* < 0.0001). The data on the AUC, the sensitivity, and the specificity are reported in Table 4.

**Table 4:** **Area under the curve (AUC), sensitivity and specificity of miR-148a-3p, miR-370-3p, and hv1-miR-B6-5p**

Comparison	AUC	95% CI	Cut-off	Sensitivity (%)	Specificity (%)
miR-148a-3p	0.665	0.5410–0.7896	0.407	80.00	51.43
miR-370-3p	0.9116	0.8452–0.9779	2.466	80.95	85.71
hv1-miR-B6-5p	0.8061	0.7054–0.9069	1.278	77.14	71.43

### Target prediction and pathway enrichment

**Figure 3:**
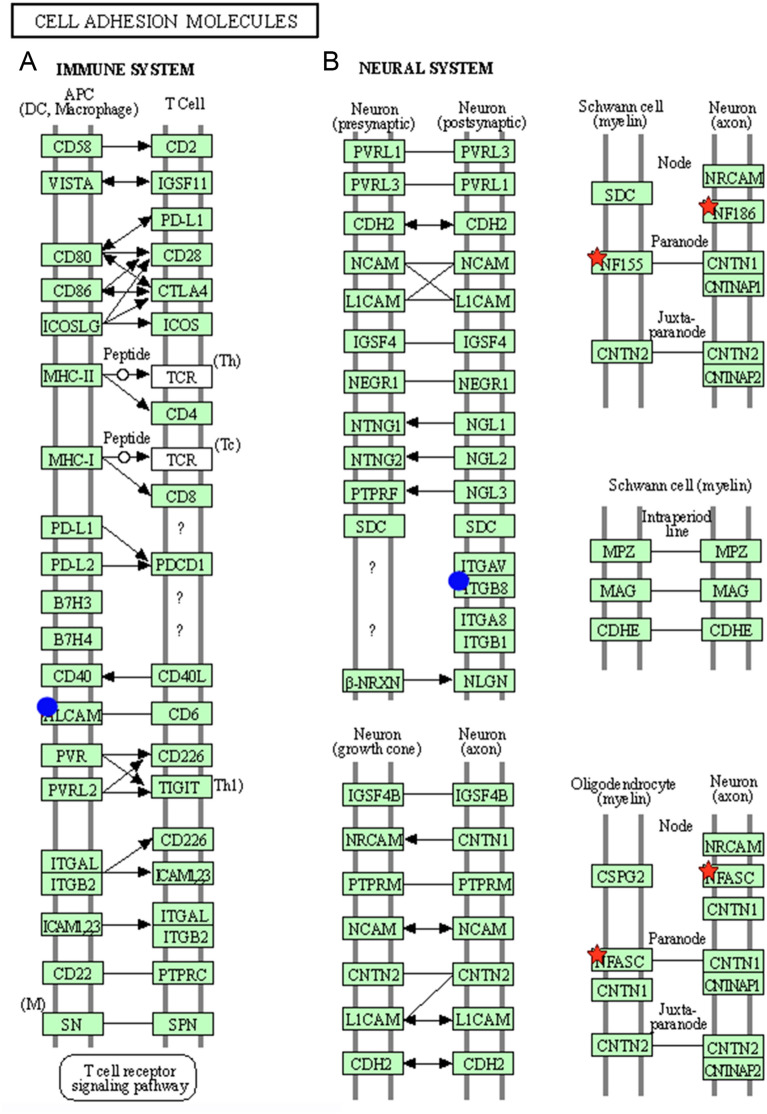
**Cell adhesion molecular pathways were identified by KEGG pathway analysis using the potential mRNA targets for differentially expressed microRNAs (DE-miRNAs) pointed out by miRWalk.** A Immune system pathway; B Neural system pathway. Proteins coded by mRNA target genes potentially modulated by miR-148a-3p are highlighted by a blue dot, while by miR-370-3p by a red star. ALCAM = activated leukocyte cell adhesion molecule; ITGB8 = Integrin Subunit Beta 8; NF and NFASC = neurofascin. The lines represent membrane bilayers, each green rectangle is a protein (a receptor or a ligand), and arrows indicate the direction of the trigger signal.

Predicted mRNA targets of DE-miRNAs were computationally retrieved from miRWalk resources. The mRNA enrichment was performed using the DAVID bioinformatics tool. The predicted mRNA targets of miR-148a-3p were 16 (14 at 3' untranslated region (UTR), 2 at 5'UTR, and 0 at coding sequence (CDS)); the predicted mRNA targets of miR-370-3p were 12 (6 at 3'UTR, 0 at 5'UTR and 6 at CDS) (Table 5). KEGG pathway analysis was performed using DAVID, pointing out that 3 pathways were significantly enriched: MAPK (Mitogen-Activated Protein Kinase) signalling pathway, cell adhesion molecules, and focal adhesion. Focusing on the cell adhesion pathway, miR-148a-3p modulates the T cell receptor signalling pathway by regulating the *ALCAM* (activated leukocyte cell adhesion molecule) gene that encodes for a protein able to bind CD6 on T cells. The neuronal system influences the expression of the *ITGB8* (Integrin Subunit Beta 8) gene encoding for a protein able to promote pre-and post-synaptic neuron interaction (Figure 3). MiR-370-3p regulates the expression of neurofascin genes (*NFASC*, *NF155,* and *NF186*) involved in Schwann cell-neuron and oligodendrocyte-neuron interactions (Figure 3).

**Table 5:** **Target genes of differentially expressed miRNAs identified using miRWalk**

Genes targeted by miR-148a-3p	Genes targeted by miR-370-3p
*ALCAM, ARL6IP1, ARRDC3, BMP3, DSTYK, DYRK1A, FXR1, ITGB8, JARID2, LBR, MAP3K4, MET, OBI1, PRNP, QKI, ZFYVE26*	*BAG4, CYB561D1, GARRE1, JARID2, LIN28A, NF155, NF186, NFASC, NSUN4, PARVB, RAD54L2, TGFBR2*

**Figure 4:**
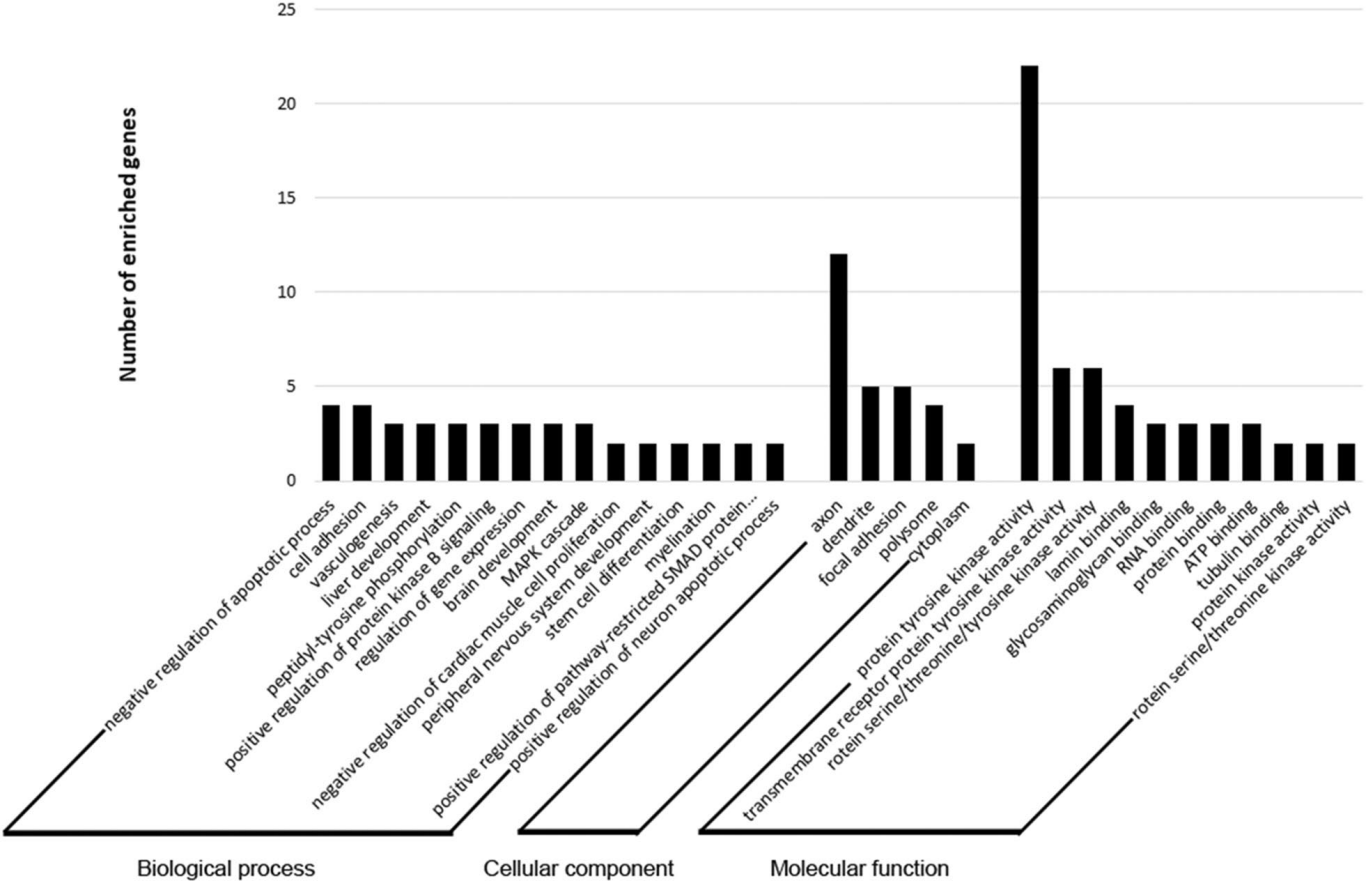
**Enriched gene ontology (GO) of terms potentially regulated by DE-miRNAs.** Target genes were annotated by DAVID at three levels: biological process, cellular component, and molecular function.

Gene Ontology (GO) analysis was performed to understand further the regulatory functions associated with the DE-miRNAs. GO enrichment analysis included the molecular function (MF), cellular component (CC), and biological process (BP) categories (Figure 4). Most MF items mainly included genes regulating protein kinase activity and binding; the enriched CC converged on genes associated with axon, dendrite, focal adhesion, polysome, and cytoplasm, BP on the apoptotic process, cell adhesion, brain development, and myelination.

## Discussion

The findings of the current study provide for the first time evidence that (a) miRNAs produced by host and BuHV-1 can be efficiently extracted and quantified by RT-qPCR from the nasal secretion of water buffaloes; (b) the expression levels of both host and BuHV-1 miRNAs between vaccinated and control water buffaloes were different; c) miR-370-3p discriminated vaccinated and control animals with excellent diagnostic accuracy. We also demonstrate that the sequences of hv1-miR-B6-5p and hv1-miR-B9 were conserved between BoHV-1 and BuHV-1.

Given that the diagnosis of herpesvirus-1 respiratory tract infection involves isolating the herpesvirus from nasal secretions [31], this matrix was selected as a potential source of miRNAs to investigate the relationship between BuHV-1 infection and host immune response at the nasal mucosa level.

This study demonstrated that the amount of hv1-miR-B6-5p decreased in vaccinated animals. The precise function of this herpesvirus miRNA is unknown. The bhv1-mir-B6-1 is antisense to intron 3 of the gene encoding BoHV-1 Infected Cell Protein 0 (bICP0) [9]. During Herpes simplex virus 1 infection, ICP0 interacts with proteins involved in the ubiquitin pathway, inactivating host intrinsic defences and hampering the infection progression [32]. Further molecular studies are required to understand the impact of vaccination on this critical region of the BoHV-1 genome via the differential expression of bhv1-miR-B6-5p. Herpesvirus particles enter the peripheral nervous system via cell-to-cell spreading. Thus, cell adhesion molecules play a pivotal role in infection [33]. The current findings suggest that miRNAs in nasal secretion might interact with pathways involved in the cell adhesion molecules pathways in the immune and neural systems. The high concentration of miR-148a-3p in vaccinated water buffaloes may inhibit the expression of *ALCAM* (activated leukocyte cell adhesion molecule or CD166) over the surface of antigen-presenting cells (APC). ALCAM, a member of the immunoglobulin superfamily expressed in endothelial and epithelial cells and on antigen-presenting cells (APCs), is the ligand of CD6, a costimulatory membrane glycoprotein involved in autoimmune and inflammatory diseases. ALCAM/CD6 binding is involved in the fine-tuning of the T cell activation and trafficking, promoting cell adhesion at the immunological synapse, lengthening the time of APC-T cell interaction, enhancing TCR signalling and MAPK pathway involved in T cell activation, proliferation, and differentiation [34, 35]. We hypothesize that since the vaccination protected animals from buHV infection, the activation of cytotoxic T cells is unnecessary, and the overexpression of miR-148a-3p downregulates the expression of ALCAM. On the other hand, buHV-1 infection may stimulate the activation of cytotoxic T cells in the nonvaccinated animals by epigenetically promoting *ALCAM* overexpression*.*

Intranasal infection of herpesvirus-1 promotes invasion of peripheral nerves reaching the central nervous system and latency in sensory neurons in the trigeminal ganglia. MiR-148a-3p and miR-370-3p potentially targeted genes coding for proteins involved in neuronal interaction. Several herpesviruses cause demyelination through direct lysis of oligodendrocytes and lysis of oligodendrocytes by stimulating the host immune response [33]. Gene Ontology analysis pointed out that miR-370-3p may regulate the expression of *NF* (neurofascin) genes, including glial NF155, neuronal NF186, and NFASC, nodal and paranodal proteins that play an important role in promoting the propagation of electrical impulses along axons between nodes of Ranvier. NFs is an L1-family immunoglobulin cell adhesion molecule that attaches myelin to axons; the thickness of the myelin sheath and the morphology of electrogenic nodes of Ranvier influence the velocity of impulse transmission along axons [36]. Since miR-370-3p affects the translation into proteins of mRNA coding for NFs, we hypothesize that in vaccinated animals, where BuHV-1 cannot spread to the sensory neurons of the peripheral nervous system and retrogradely towards the trigeminal ganglia, the low level of miR-370-3p does not interfere with NF genes allowing the normal function of neurons. At the same time, the overexpression of this miRNA may promote in control nonvaccinated animals the demyelination of neurons, as reported for herpes simplex virus type 1 (HSV-1) [37, 38]. Similarly, a low level of miR-370-3p, which is also associated with the modulation of inflammation by inhibiting ROS production by targeting TLR4 [39], oxidative stress, and apoptosis [40], may dampen the inflammatory response in vaccinated animals, while in control animals BuHV-1 may promote immune-suppressive activities associated with latency in sensory neurons in trigeminal ganglia [41, 42].

ROC analysis highlighted that miR-370-3p could discriminate vaccinated from control water buffaloes with high sensitivity (= 80.95%) and specificity (= 85.71%), suggesting that this miRNA may be an excellent candidate biomarker to support an in-field, rapid screening. It should be noted that the methods applied to detect microRNAs have the advantage of obtaining results quickly compared to classical virologic methods on cell cultures that require several days. Moreover, nasal swabs represent a fast and non-invasive procedure for water buffaloes because it avoids adverse effects, such as stress, and reduce the cost of diagnosing BuHV-1. In addition, they can be easily collected and tested for diagnosis of BuHV-1 outbreaks. Finally, microRNAs can be collected from field matrices, and the tests can be performed directly on the farm with the appropriate equipment and reagents.

In conclusion, the present study identified for the first time DE-miRNAs in the nasal secretion of water buffaloes affected by BuHV-1, providing a molecular basis for understanding the horizontal transmission of the BuHV-1 and the entry into the organism of BuHV-1 in water buffaloes. The DE-miRNAs regulated the transcriptions of genes related to the BuHV-1 latency and host immune response. Moreover, we found that miR-370-3p is a candidate biomarker of BuHV-1 infection. Although this study provided new and important insights into BuHV-1 infection, further experiments involving more animals are required to validate the miRNA potential use as an in-field biomarker. Additional information on the molecular mechanisms regulated by these miRNAs will promote the implementation of new interventions in the field.

### Supplementary Information

**Additional file 1. Sequences alignment.** The results of Sanger sequencing have been aligned with the sequences of the plasmid pGEM, and a) bhv1-miR-B6-5p, or b) bhv1-miR-B9. The black nucleotides are the poly-A tail and adaptors added during the reverse transcription step of miRNAs into cDNA using the kit TaqMan Advanced miRNA cDNA Synthesis Kit.

## Data Availability

The datasets used and/or analysed during the current study are available from the corresponding author upon reasonable request.

## References

[1] Amoroso MG, Corrado F, De Carlo E, Lucibelli MG, Martucciello A, Guarino A, Galiero G (2013). Bubaline herpesvirus 1 associated with abortion in a Mediterranean water buffalo. Res Vet Sci.

[2] De Carlo E, Re GN, Letteriello R, Del Vecchio V, Giordanelli MP, Magnino S, Fabbi M, Bazzocchi C, Bandi C, Galiero G (2004). Molecular characterisation of a field strain of bubaline herpesvirus isolated from buffaloes (Bubalus bubalis) after pharmacological reactivation. Vet Rec.

[3] Petrini S, König P, Righi C, Iscaro C, Pierini I, Casciari C, Pellegrini C, Gobbi P, Giammarioli M, De Mia GM (2020). Serological cross-reactivity between bovine alphaherpesvirus 2 and bovine alphaherpesvirus 1 in a gB-ELISA: a case report in Italy. Front Vet Sci.

[4] Bartel DP (2009). MicroRNAs: target recognition and regulatory functions. Cell.

[5] Saliminejad K, Khorram Khorshid HR, Soleymani Fard S, Ghaffari SH (2019). An overview of microRNAs: biology, functions, therapeutics, and analysis methods. J Cell Physiol.

[6] Umbach JL, Cullen BR (2009). The role of RNAi and microRNAs in animal virus replication and antiviral immunity. Genes Dev.

[7] Jurak I, Griffiths A, Coen DM (2011). Mammalian alphaherpesvirus miRNAs. Biochim Biophys Acta.

[8] Pfeffer S, Sewer A, Lagos-Quintana M, Sheridan R, Sander C, Grässer FA, van Dyk LF, Ho CK, Shuman S, Chien M, Russo JJ, Ju J, Randall G, Lindenbach BD, Rice CM, Simon V, Ho DD, Zavolan M, Tuschl T (2005). Identification of microRNAs of the herpesvirus family. Nat Methods.

[9] Glazov EA, Horwood PF, Assavalapsakul W, Kongsuwan K, Mitchell RW, Mitter N, Mahony TJ (2010). Characterization of microRNAs encoded by the bovine herpesvirus 1 genome. J Gen Virol.

[10] Jaber T, Workman A, Jones C (2010). Small noncoding RNAs encoded within the bovine herpesvirus 1 latency-related gene can reduce steady-state levels of infected cell protein 0 (bICP0). J Virol.

[11] Tang S, Bertke AS, Patel A, Wang K, Cohen JI, Krause PR (2008). An acutely and latently expressed herpes simplex virus 2 viral microRNA inhibits expression of ICP34.5, a viral neurovirulence factor. Proc Natl Acad Sci U S A.

[12] Tang S, Patel A, Krause PR (2009). Novel less-abundant viral microRNAs encoded by herpes simplex virus 2 latency-associated transcript and their roles in regulating ICP34.5 and ICP0 mRNAs. J Virol.

[13] Umbach JL, Kramer MF, Jurak I, Karnowski HW, Coen DM, Cullen BR (2008). MicroRNAs expressed by herpes simplex virus 1 during latent infection regulate viral mRNAs. Nature.

[14] Kanokudom S, Mahony TJ, Smith DR, Assavalapsakul W (2018). Modulation of bovine herpesvirus 1 infection by virally encoded microRNAs. Virus Res.

[15] Raaperi K, Orro T, Viltrop A (2014). Epidemiology and control of bovine herpesvirus 1 infection in Europe. Vet J.

[16] Petrini S, Martucciello A, Grandoni F, De Matteis G, Cappelli G, Giammarioli M, Scoccia E, Grassi C, Righi C, Fusco G, Galiero G, Pela M, De Mia GM, De Carlo E (2021). Evaluation of safety and efficacy of an inactivated marker vaccine against Bovine alphaherpesvirus 1 (BoHV-1) in water buffalo (Bubalus bubalis). Vaccines.

[17] Reed LJ, Muench H (1938). A simple method of estimating fifty per cent endpoints. Am J Epidemiol.

[18] Bustin SA, Benes V, Garson JA, Hellemans J, Huggett J, Kubista M, Mueller R, Nolan T, Pfaffl MW, Shipley GL, Vandesompele J, Wittwer C (2009). The MIQE Guidelines: minimum information for publication of quantitative real-time PCR experiments. Clin Chem.

[19] Howe C (2007). Gene Cloning and Manipulation.

[20] Hellemans J, Mortier G, De Paepe A, Speleman F, Vandesompele J (2008). qBase relative quantification framework and software for management and automated analysis of real-time quantitative PCR data. Genome Biol.

[21] qbase+ software. www.qbaseplus.com. Accessed 20 May 2022

[22] Sticht C, De La Torre C, Parveen A, Gretz N (2018). miRWalk: an online resource for prediction of microRNA binding sites. PLoS ONE.

[23] Lecchi C, Zamarian V, Borriello G, Galiero G, Grilli G, Caniatti M, D'Urso ES, Roccabianca P, Perego R, Minero M, Legnani S, Calogero R, Arigoni M, Ceciliani F (2020). Identification of altered miRNAs in cerumen of dogs affected by otitis externa. Front Immunol.

[24] Wong N, Wang X (2015). miRDB: an online resource for microRNA target prediction and functional annotations. Nucleic Acids Res.

[25] Hsu SD, Lin FM, Wu WY, Liang C, Huang WC, Chan WL, Tsai WT, Chen GZ, Lee CJ, Chiu CM, Chien CH, Wu MC, Huang CY, Tsou AP, Huang HD (2011). miRTarBase: a database curates experimentally validated microRNA–target interactions. Nucleic Acids Res.

[26] Agarwal V, Bell GW, Nam J-W, Bartel DP (2015). Predicting effective microRNA target sites in mammalian mRNAs. Elife.

[27] Huang DW, Sherman BT, Lempicki RA (2009). Bioinformatics enrichment tools: paths toward the comprehensive functional analysis of large gene lists. Nucleic Acids Res.

[28] Huang DW, Sherman BT, Lempicki RA (2009). Systematic and integrative analysis of large gene lists using DAVID bioinformatics resources. Nat Protoc.

[29] Kanehisa M, Goto S, Sato Y, Furumichi M, Tanabe M (2012). KEGG for integration and interpretation of large-scale molecular data sets. Nucleic Acids Res.

[30] Zou KH, O’Malley AJ, Mauri L (2007). Receiver-operating characteristic analysis for evaluating diagnostic tests and predictive models. Circulation.

[31] Kahrs RF (1977). Infectious bovine rhinotracheitis: a review and update. J Am Vet Med Assoc.

[32] Rodríguez MC, Dybas JM, Hughes J, Weitzman MD, Boutell C (2020). The HSV-1 ubiquitin ligase ICP0: modifying the cellular proteome to promote infection. Virus Res.

[33] Jones C (2019). Bovine herpesvirus 1 counteracts immune responses and immune-surveillance to enhance pathogenesis and virus transmission. Front Immunol.

[34] Santos FR, Oliveira L, Carmo MA (2016). Tuning T cell activation: the function of CD6 at the immunological synapse and in T cell responses. Curr Drug Targets.

[35] te Riet J, Helenius J, Strohmeyer N, Cambi A, Figdor CG, Müller DJ (2014). Dynamic coupling of ALCAM to the actin cortex strengthens cell adhesion to CD6. J Cell Sci.

[36] Dutta DJ, Woo DH, Lee PR, Pajevic S, Bukalo O, Huffman WC, Wake H, Basser PJ, SheikhBahaei S, Lazarevic V, Smith JC, Fields RD (2018). Regulation of myelin structure and conduction velocity by perinodal astrocytes. Proc Natl Acad Sci U S A.

[37] Bello-Morales R, Andreu S, López-Guerrero JA (2020). The role of herpes simplex virus type 1 infection in demyelination of the central nervous system. Int J Mol Sci.

[38] Bello-Morales R, Andreu S, Ripa I, López-Guerrero JA (2021). HSV-1 and endogenous retroviruses as risk factors in demyelination. Int J Mol Sci.

[39] Tian D, Sha Y, Lu J, Du X (2018). MiR-370 inhibits vascular inflammation and oxidative stress triggered by oxidized low-density lipoprotein through targeting TLR4. J Cell Biochem.

[40] Qiu Z, Wang L, Mao H, Xu F, Sun B, Lian X, Wang J, Kong F, Wang L, Chen Y (2019). MiR-370 inhibits the oxidative stress and apoptosis of cardiac myocytes induced by hydrogen peroxide by targeting FOXO1. Exp Ther Med.

[41] Jones C (2013). Bovine herpes virus 1 (BHV-1) and herpes simplex virus type 1 (HSV-1) promote survival of latently infected sensory neurons, in part by inhibiting apoptosis. J Cell Death.

[42] Jones C, da Silva LF, Sinani D (2011). Regulation of the latency–reactivation cycle by products encoded by the bovine herpesvirus 1 (BHV-1) latency-related gene. J Neurovirol.

