# Supplementary figures and images for: Endogenous and viral microRNAs in nasal secretions of water buffaloes (Bubalus bubalis) after Bubaline alphaherpesvirus 1 (BuHV-1) challenge infection
Source: Vet Res. 2023 Jun 5;54:44. doi: 10.1186/s13567-023-01175-9 (PMC10242922; doi:10.1186/s13567-023-01175-9)

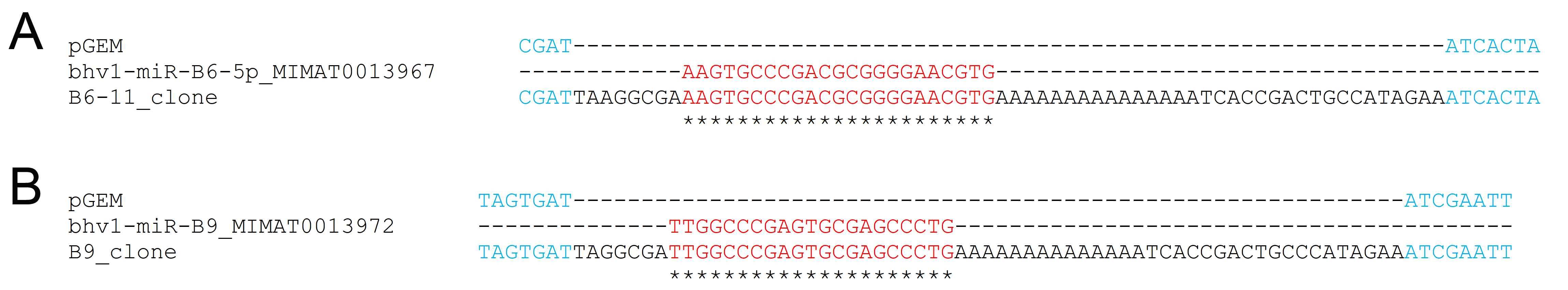

Supplement: Supplementary file 1 — Additional file 1. Sequences alignment. The results of Sanger sequencing have been aligned with the sequences of the plasmid pGEM, and a) bhv1-miR-B6-5p, or b) bhv1-miR-B9. The black nucleotides are the poly-A tail and adaptors added during the reverse transcription step of miRNAs into cDNA using the kit TaqMan Advanced miRNA cDNA Synthesis Kit. [file 13567_2023_1175_MOESM1_ESM.docx]
